# Comparison of risk assessment in 1652 early ER positive, HER2 negative breast cancer in a real-world data set: classical pathological parameters vs. 12-gene molecular assay (EndoPredict)

**DOI:** 10.1007/s10549-021-06415-0

**Published:** 2021-11-16

**Authors:** Paul Jank, Judith Lea Lindner, Annika Lehmann, Berit Maria Pfitzner, Jens-Uwe Blohmer, David Horst, Ralf Kronenwett, Carsten Denkert, Wolfgang Daniel Schmitt

**Affiliations:** 1 Institute of Pathology, Philipps-University Marburg and University Hospital Marburg (UKGM)—Universitätsklinikum Marburg, Baldingerstraße, 35043 Marburg, Germany; 2 Charité – Universitätsmedizin Berlin, Corporate member of Freie Universität Berlin and Humboldt Universität zu Berlin, Institute of Pathology, Charitéplatz 1, 10117 Berlin, Germany; 3 Institute of Pathology, DRK Hospital Berlin Westend, Berlin, Germany; 4 Charité – Universitätsmedizin Berlin, Corporate member of Freie Universität Berlin and Humboldt Universität zu Berlin, Department of Gynecology and Obstetrics, Charitéplatz 1, 10117 Berlin, Germany; 5 Myriad International GmbH, Cologne, Germany

**Keywords:** Breast cancer, Chemotherapy, Endocrine therapy, Molecular score, Risk assessment

## Abstract

**Background:**

Risk assessment on the molecular level is important in predictive pathology to determine the risk of metastatic disease for ERpos, HER2neg breast cancer. The gene expression test EndoPredict (EP) was trained and validated for prediction of a 10-year risk of distant recurrence to support therapy decisions regarding endocrine therapy alone or in combination with chemotherapy. The EP test provides the 12-gene Molecular Score (MS) and the EPclin-Score (EPclin), which combines the molecular score with tumor size and nodal status. In this project we investigated the correlation of 12-gene MS and EPclin scores with classical pathological markers.

**Methods:**

EndoPredict-based gene expression profiling was performed prospectively in a total of 1652 patients between 2017 and 2020. We investigated tumor grading and Ki67 cut-offs of 20% for binary classification as well as 10% and 30% for three classes (low, intermediate, high), based on national and international guidelines.

**Results:**

410 (24.8%) of 1652 patients were classified as 12-gene MS low risk and 626 (37.9%) as EPclin low risk. We found significant positive associations between 12-gene MS and grading (*p* < 0.001), EPclin and grading (*p* = 0.001), 12-gene MS and Ki67 (*p* < 0.001), and EPclin and Ki67 (*p* < 0.001). However, clinically relevant differences between EP test results, Ki67 and tumor grading were observed. For example, 118 (26.3%) of 449 patients with Ki67 > 20% were classified as low risk by EPclin. Same differences were seen comparing EP test results and tumor grading.

**Conclusion:**

In this study we could show that EP risk scores are distributed differentially among Ki67 expression groups, especially in Ki67 low and high tumors with a substantial proportion of patients with EPclin high risk results in Ki67 low tumors and vice versa. This suggests that classical pathological parameters and gene expression parameters are not interchangeable, but should be used in combination for risk assessment.

**Supplementary Information:**

The online version contains supplementary material available at 10.1007/s10549-021-06415-0.

## Introduction

For patients with estrogen receptor positive (ER pos), human epidermal growth factor receptor 2 negative (HER2 neg) breast cancer the most challenging diagnostic decision is the separation of patients with low recurrence risk, who would benefit from endocrine therapy (ET) alone from those with high recurrence risk, who benefit from addition of chemotherapy (CTx) to ET. Gene expression profiling is a central element of the diagnostic workup for risk assessment and the current ESMO guidelines recommend the addition of genetic assays to complement pathological assessment including tumor burden, tumor grading, tumor proliferation, and vascular invasion [1].

The EndoPredict test (EP) is a mRNA based 12-gene molecular assay to determine the 10-year recurrence risk in an adjuvant setting of ER pos, HER2 neg breast cancer. The EP test is used in clinical routine diagnostic to identify patients with low versus high recurrence risk to support therapy decisions regarding endocrine therapy alone or in combination with chemotherapy. The EP test provides the 12-gene MS score using quantitative real-time RT-PCR. This biological score is combined with staging parameters like tumor size and nodal status to generate the EPclin score, which is the basis for determination of 10-year recurrence risk. [2].

As part of the prospective-retrospective validation in clinical trial cohorts, it was shown that EP could identify more patients as low risk compared with classical pathological parameters and can help to reduce chemotherapy in low risk ER pos, HER2 neg breast cancer patients [3–7]. As a general strategy, it is recommended to use gene expression profiling (molecular testing) in patients with an intermediate risk based on clinicopathological parameters, however, the definition of this intermediate risk groups is variable.

International guidelines describe intermediate risk group based on Ki67 (10.1–30% or 5.1–30%) or online tools like “PREDICT” for molecular testing for decision for adjuvant systemic chemotherapy [1, 8–12].

German criteria for reimbursement for EP testing in specialized centers (ASV criteria) for primary ER pos, HER2 neg BC in adjuvant settings recommend molecular gene expression assays for grade 2 (G2) tumors but not Ki67 > 30%, or for tumors with a Ki67 between 10 and 30% but not grade 3 (G3) for node negative (N0) patients and grade 1 (G1) or G2 tumors or tumors with Ki67 between 10 and 30% but not G3 or Ki67 > 30% in node-positive (1–3 positive lymph nodes) patients. Suppl. Table 1 summarizes international clinical guidelines regarding breast cancer risk groups, based on Ki67 and use of molecular testing for adjuvant systemic chemotherapy. These suggestions are based on the general adoption that gene expression assays should be focused on an intermediate risk patient group, however, the distribution of gene expression-based risk groups in patient groups defined by classical pathology parameters is not known. As shown, there is a lack of global, evidence based clinical guidelines regarding molecular testing and/or Ki67’s role for individual risk assessment in adjuvant setting.

The aim of this study is the comparison of the classical pathological parameters grading and Ki67 with gene-expression-based risks groups in a large consecutive cohort of 1652 patients from clinical routine diagnostic.

## Materials and methods

### Study population and clinicopathological parameters

The EndoPredict gene expression assay (Myriad International GmbH, Cologne, Germany) was performed prospectively as part of routine clinical workup between July 2017 and June 2020 at the Institute of Pathology, Charité—Universitätsmedizin Berlin. For 1652 (76.41%) of 2162 patients, tumor grading and proliferation (Ki67) at time of diagnosis or at surgery were assessed as part of local pathological examination and were available for this analysis. To classify individual recurrence risks, we used two Ki-67 classifications: According to St. Gallen guidelines 2013, Ki67 cut-offs were set at ≤ 20% for Ki67 low and > 20% for Ki-67 high, for binary classification [13–15]. Cut-offs defined by national German regulations for clinical use of gene expression assays (developed by the German Federal Joint Committee (G-BA)) were used to generate a variable with three groups: low Ki-67 (≤ 10%), intermediate Ki67 (10.1–30.0%), and high Ki67 (> 30%). To compare our results with international guidelines for risk assessment in early ER pos, HER2 neg BC, we displayed our results with international Ki67 cut-offs in Suppl. Figure 1. Since this study is based on a real-world data set, Ki67 and tumor grading were reported from local pathologies, without central pathology assessment.

Table 1 gives an overview of the baseline characteristics of the cohort.

**Table 1:** Baseline parameters in study cohort (*N* = 1652)

	Category	Frequency N(%)	Mean	95% CI (mean)
Overall study cohort		1652 (100.0)		
12-gene MS	Low (< 5.0)	410 (24.8)	6.664	6.55–6.78
	High (≥ 5.0)	1242 (75.2)		
EPclin	Low (< 3.3)	626 (37.9)	3.593	3.56–3.63
	High (≥ 3.3)	1026 (62.1)		
Ki-67 two groups	Low (≤ 20%)	1203 (72.8)	17.45	16.96–17.94
	High (> 20%)	449 (27.2)		
Ki-67 three groups	Low (≤ 10%)	557 (33.7)	17.45	16.96–17.94
	Intermediate (10.1 – 30.0%)	994 (60.2)		
	High (> 30%)	101 (6.1)		
Grading	G1	140 (8.5)	2.03	N/A
	G2	1328 (80.4)		
	G3	184 (11.1)		

The diagnostic assessment was performed as part of the clinical routine. The evaluation of existing diagnostic data was performed according to the Berlin hospital law. Ethical approval was given by the ethics committee of Charité Universitätsmedizin Berlin (EA/074/18).

### Assessment of the EndoPredict test and score

EndoPredict tests were performed based on the standardized EndoPredict protocol according to manufacturer’s instructions.

The 12-gene MS incorporates the expression of eight cancer-related genes (*STC2*, *UBE2C*, *BIRC5*, *RBBP8*, *DHCR7*, *IL6ST*, *AZGP1*, and *MGP*), three housekeeping genes (*CALM2*, *OAZ1*, and *RPL37A*), and one control gene (*HBB*) [2, 16]. EPclin score, based on 12-gene MS, additionally takes tumor staging and nodal status into account.

Based on the predefined cut points, tumors with 12-gene MS < 5.0 were classified as low risk and ≥ 5.0 as high risk. Including tumor staging and nodal status, the predefined EPclin score risk groups were low risk (< 3.32867) and high risk (≥ 3.32867).

### Statistical methods

The statistical analysis was conducted using SPSS Statistics Version 25 (IBM, Armonk, USA). For categorical variables we determined *p* values with Fisher’s exact test in 2 × 2 matrix. For matrices > 2 × 2 the chi-square test was used. For mean comparison a two-sided students t-test was used to determine the *p* value. For confidence intervals (CI) 95% were used. Statistically significant cases were at a p-value ≤ 0.050. A statistical analysis plan (SAP) was finalized prior to data review and analysis. SAP did not include Bonferroni correction for multiple testing.

## Results

### Baseline characteristics of study cohort

As shown in Table 1, we evaluated EP test results in a total of 1652 patients, tested from 2017 to 2020. In 410 (24.8%) of patients we detected a 12-gene MS low score, while an EPclin low score was found in 626 (37.9%) of all patients. The mean 12-gene MS was 6.664 (95%CI 6.55–6.78) and mean EPclin was 3.593 (95%CI 3.56–3.63). Using Ki67 variable with three groups (low, intermediate, high), 557 (33.7%) of 1652 patients were in the Ki67 low (≤ 10%) group, 994 (60.2%) in the Ki67 intermediate group (10.1–30%), and 101 (6.1%) in the Ki67 high group (> 30%). Mean Ki-67 expression was 17.45% (95%CI 16.96–17.94). Using Ki67 variable with two groups, 1203 (72.8%) of 1652 patients were in the Ki67 low (≤ 20%) group and 449 (27.2%) patients in the Ki67 high (> 20%) group. 1328 (80.4%) of 1652 tumors were classified as moderately differentiated (G2), followed by G3 with 184 (11.1%) cases and G1 with 140 (8.5%) tumors.

### Distribution of EP scores among tumor proliferation and tumor grading

**Fig.1:**
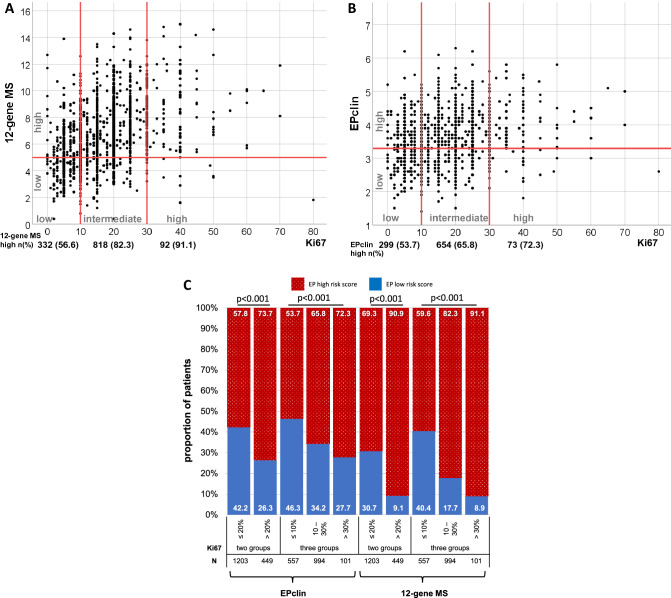
Distribution of 12-gene MS and EPclin scores and low/high risk groups among Ki67 groups and as continuous variable. **A**, **B** Proportion of patients in risk score groups stratified by Ki67 three groups. **C** 12-gene MS and EPclin versus Ki67 as continuous variable. Numbers in bars are percentage of each group. *p* values determined by two-sided χ^2^test

A graphical depiction of continuous 12-gene MS and EPclin show a slight positive association with continuous Ki67 expression, as shown in Fig. 1A and B. Furthermore, a significant statistical correlation between classical pathological parameters and EP test results was found: Our analysis shows significances with *p* values *p* < 0.001 (EP and grading), *p* = 0.001 (EPclin and grading), *p* < 0.001 (EP and Ki67), and *p* < 0.001 (EPclin and Ki67). Beside these strong associations, there were relevant discordances between EP scores and clinical parameters: As shown in Fig. 1C, in low proliferating tumors with Ki67 ≤ 20%, 695 (57.8%) of 1203 patients had a EPclin high risk score. In high proliferating tumors (Ki67 > 20%) 118 (26.3%) of 449 patients had a EPclin low risk score. Similarly, in the Ki67 low (≤ 10%) group 299 (53.7%) of 557 patients had EPclin high results and in the Ki67 high (> 30%) group 28 (27.7%) of 101 patients were classified as EPclin low. To compare the proportion of Ki67 low/high in EP risk groups with other publications we evaluated these percentages in Suppl. Table 2.

81 (57.9%) of 140 patients with grade 1 tumors had a EPclin high risk score. In comparison, in 46 (25.0%) of 184 G3 cases showed low EPclin risk score. Similar results were seen using 12-gene-MS.

**Fig.2:**
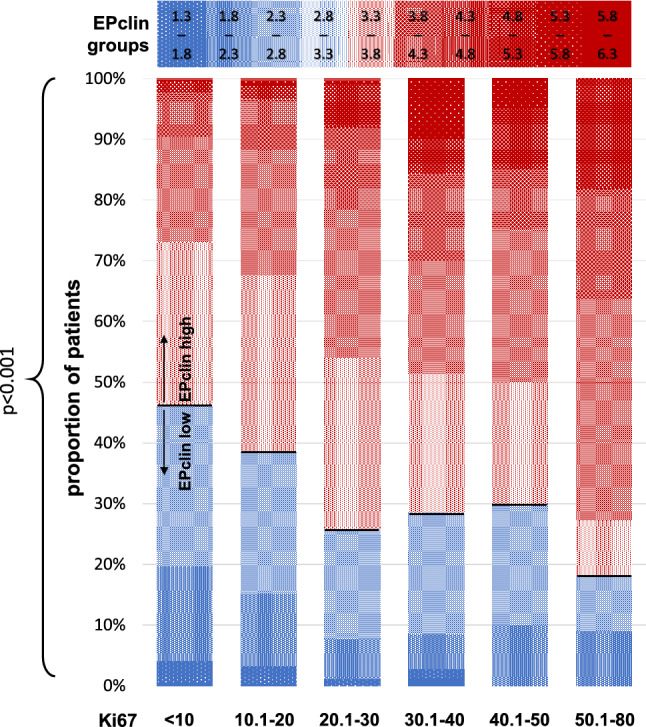
Grouped EPclin distribution in Ki67 groups. Proportion of low risk patients are marked in blue, proportion of high risk scores are marked in red. EPclin groups estimated from EPclin cut-off 3.32867

Figure 2 illustrates proportions of EPclin groups (starts from exact cut-off 3.32867 in 0.5 up and down) in Ki67 groups (in 10% steps). For 12-gene MS mean score in G1 cohort was 5.64 (95%CI 5.31–5.96), in G2 cohort mean was 6.56 (95%CI 6.44–6.81), and in G3 cohort mean was 8.19 (95%CI 7.85–8.54). All means are statistically significant different with *p* < 0.001. For EPclin mean score in G1 cohort was 3.50 (95%CI 3.40–3.61), in G2 cohort mean was 3.56 (95%CI 3.53–3.61), and in G3 cohort mean was 3.87 (95%CI 3.76–4.00). Between G1 and G2 cohort means were not significantly different (*p* = 0.324). Between G1 and G3 cohort and G2 and G3 cohort means were significantly different with *p* < 0.001.

We observed an increasing of EPclin high risk score with increasing Ki67 status, as shown in Fig. 1C (*p* < 0.001). Boxplots for continuous parameters in comparison with EP test results are shown in supplemental material as well as cross tables with all parameters shown in this analysis.

## Discussion

Our study demonstrates that both EP risk scores are generally associated with tumor proliferation and tumor grading. However, a clinically relevant amount of cases show a discordance of clinical and molecular risk parameters. For example, patients with classical pathological low risk factors, e.g., in Ki67 ≤ 10% (53.7% of patients) or ≤ 20% (57.8% of patients) groups, can still have a EPclin high risk—same results were shown using 12-gene MS. Similar results occurred in aggressive tumors with high Ki67 > 30% (27.7% of patients) and high grade (G3, 25.0% of patients)—here we detected a remarkable amount of cases with 12-gene MS and/or EPclin low risk results. These discordances could be relevant in clinical use to determine those patients who are classically low risk but show a high EP test result and may benefit from addition of CTx.

Since molecular tests are more reproducible and quantitative than classical methods molecular tests might be used in addition to classical factors in an intermediate recurrence risk group to get a more precise risk assessment. This is supported by previous studies showing that the combination of clinical and molecular information enhanced prognostic performance of gene expression assays [2, 17]. Prediction of early and late recurrence is primarily driven by the two biological motives proliferation and estrogen receptor signaling and the two clinical factors tumor size and nodal status [2, 4, 17]. Previous analyses have also shown that EndoPredict provided prognostic power independent from subtype defined by Ki67 [18].

Further investigations are needed, to determine the relevance of this discrepant risk assessment results and its clinical importance. A comparison of performance of six prognostic signatures with predefined cutoffs for ER pos, HER2 neg BC from Sestak et al. showed a high correlation of EP test results with recurrence after 10 years. For patients with node-positive, ER pos, HER2 neg disease, EPclin predicted the 10-year recurrence risk with a hazard ratio (HR) of 1.69 [17]. This study also showed that gene expression tests that include clinical factors like tumor size and nodal status in their test result had a better prognostic power than those without including clinical factors supporting that molecular information should be used in addition to classical clinical factors in order to get a more accurate risk assessment.

We compared the proportion of patients with low and high Ki67 expression in EPclin risk groups from other publications with our results, see Suppl. Table 2.

Noske et al. published similar results in a cohort of 307 luminal breast cancers. The authors conclude that Ki67 is statistically significantly correlated (*p* < 0.001) with EP test results, as confirmed also in our study. They also noticed discordant cases as in our study. [19].

Interestingly, Pelliccia et al. published their results to compare risk prediction, using Ki67 expression and cutoff at 30%, with EP test results: In a cohort of 100 patients 28 were classified as Ki67 high risk, 24 of these were EPclin high risk, and 4 EPclin low risk. In the Ki67 low risk group (*N* = 21), one patient was determined as EPclin high risk and 20 as EPclin low risk. [20].

Almsted et al. investigated the influence of clinicopathological factors on the use of the EndoPredict assay. 156 accomplished EndoPredict assays were also compared with patient and tumor characteristics and show similar results regarding EP test result distribution among Ki67 and grading classes. They also showed noteworthy discordances for low grade tumors and high EP test results and vice versa. The proportion of EPclin high risk tumors in the Ki67 low (≤ 20%) group is nearly 47% being close to our results (42.2%) [21].

To underline discrepancies in risk assessment, we also stratified the proportion of patients with EPclin low and high risk from our study by actual Ki67 cutoffs from international guidelines, summarized in Suppl. Figure 3.

Ettl and co-authors reported similar results regarding EP test scores and tumor grading: In a dataset of 373 patients, 60 (16.1%) patients with grade 1 tumors were EPclin low and 10 (2.7%) EPclin high risk. On the other hand, grade 2 tumors (*N* = 46) were classified as EPclin low in 63 cases and EPclin high risk in 46 tumors [22].

## Conclusion

In conclusion, classical pathological parameters (Ki67 and tumor grading) correlate statistically significantly (*p* < 0.001) with 12-gene MS and EPclin risk scores. Despite this high correlation, a relevant number of cases show a discordance of clinicopathological and molecular risk parameters. This suggests that these two approaches are not interchangeable and should be used together for an adequate therapy decision (ET alone or in combination with CTx) in primary ER pos, HER2 neg breast cancer. This suggests that classical pathological parameters and gene expression parameters are not interchangeable, but should be used in combination for risk assessment, for each individual patient. In future projects the different clinical outcome of different patient subgroups should be brought into focus.

## Supplementary Information

Below is the link to the electronic supplementary material.

Supplementary file1 (PPTX 68 kb)

Supplementary file2 (DOCX 46 kb)

## Data Availability

All relevant data are within the paper and its Supporting Information files. The data underlying the results presented in the study are available from the study group, some restriction apply due to confidentiality of patient data. Since these data are derived from a prospective research trial with ongoing follow up there are legal and ethical restrictions to share sensitive patient related data publicly. Data can be requested in context of a translational research project by sending a request to the corresponding author.

## Abbreviations

12-gene MS: 12-Gene molecuar score
BC: Breast cancer
CI: Confidence interval
CTx: Chemotherapy
EP: EndoPredict
EPclin: Combination of 12-gene MS with staging and nodal status
ER: Estrogen receptor
ET: Endocrine therapy
G1: Grade 1
G2: Grade 2
G3: Grade 3
HER2: Human epidermal growth factor receptor 2
HR: Hazard ratio
