# Supplementary material for: Comparison of risk assessment in 1652 early ER positive, HER2 negative breast cancer in a real-world data set: classical pathological parameters vs. 12-gene molecular assay (EndoPredict)
Source: Breast Cancer Res Treat. 2021 Nov 16;191(2):327–33. doi: 10.1007/s10549-021-06415-0 (PMC8763835; doi:10.1007/s10549-021-06415-0)
Supplement: Supplementary file 1 — Supplementary file1 (PPTX 68 kb) [file 10549_2021_6415_MOESM1_ESM.pptx]

## Slide 1
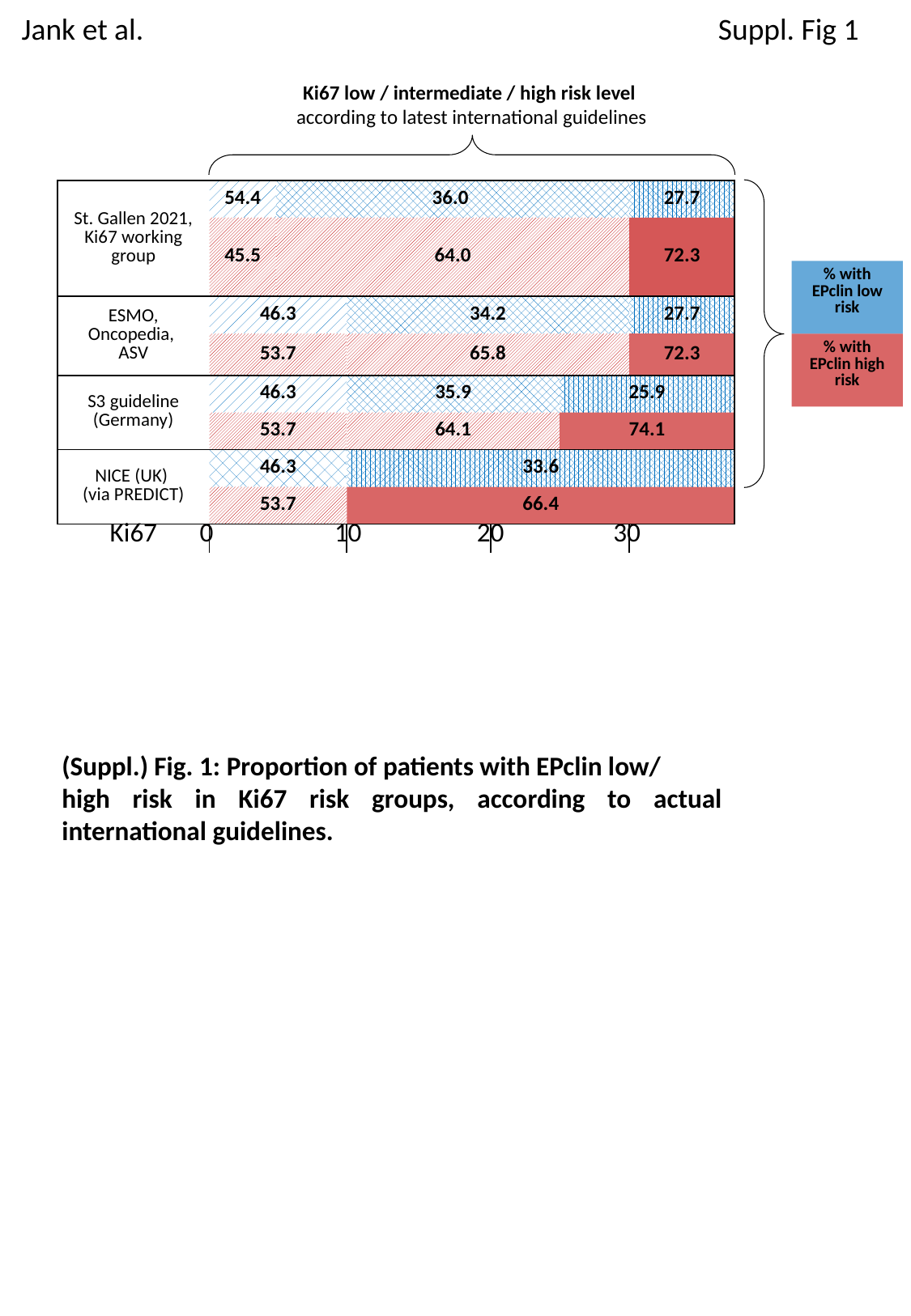

Jank et al. 				 Suppl. Fig 1
Ki67 low / intermediate / high risk level
according to latest international guidelines
| St. Gallen 2021, Ki67 working group | 54.4 | 36.0 | | | | 27.7 |
| --- | --- | --- | --- | --- | --- | --- |
| | 45.5 | 64.0 | | | | 72.3 |
| ESMO, Oncopedia, ASV | 46.3 | | 34.2 | | | 27.7 |
| | 53.7 | | 65.8 | | | 72.3 |
| S3 guideline (Germany) | 46.3 | | 35.9 | | 25.9 | |
| | 53.7 | | 64.1 | | 74.1 | |
| NICE (UK) (via PREDICT) | 46.3 | | 33.6 | | | |
| | 53.7 | | 66.4 | | | |
| | | | | | | |
| % with EPclin low risk |
| --- |
| % with EPclin high risk |
Ki67 0 10 20 30
(Suppl.) Fig. 1: Proportion of patients with EPclin low/
high risk in Ki67 risk groups, according to actual international guidelines.

## Slide 2
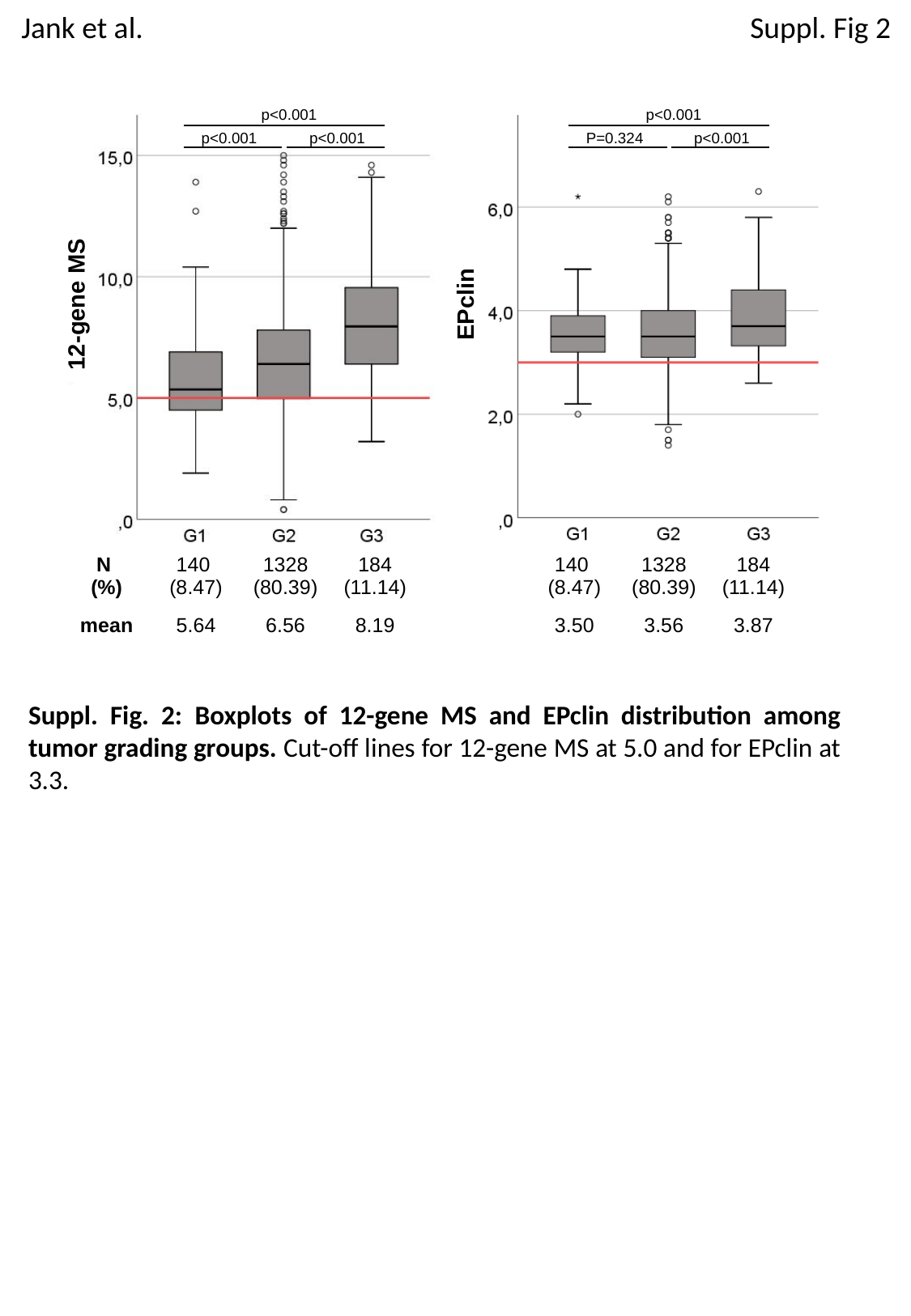

Jank et al. 					Suppl. Fig 2
p<0.001
p<0.001
p<0.001
p<0.001
P=0.324
p<0.001
12-gene MS
EPclin
| N (%) | 140 (8.47) | 1328 (80.39) | 184 (11.14) |
| --- | --- | --- | --- |
| mean | 5.64 | 6.56 | 8.19 |
| | 140 (8.47) | 1328 (80.39) | 184 (11.14) |
| --- | --- | --- | --- |
| | 3.50 | 3.56 | 3.87 |
Suppl. Fig. 2: Boxplots of 12-gene MS and EPclin distribution among tumor grading groups. Cut-off lines for 12-gene MS at 5.0 and for EPclin at 3.3.

## Slide 3
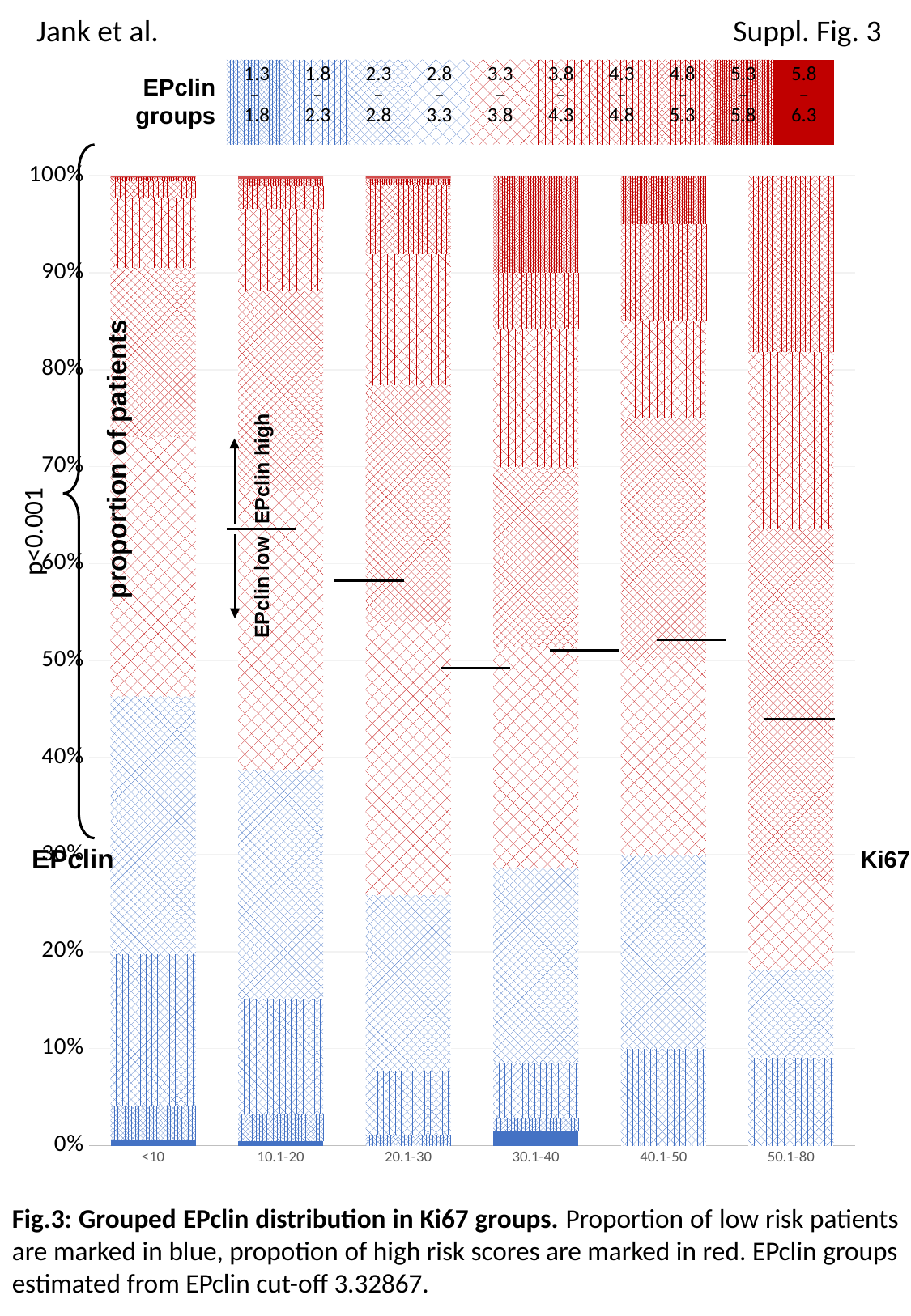

Jank et al. 				 Suppl. Fig. 3
| 1.3 – 1.8 | 1.8 – 2.3 | 2.3 – 2.8 | 2.8 – 3.3 | 3.3 – 3.8 | 3.8 – 4.3 | 4.3 – 4.8 | 4.8 – 5.3 | 5.3 – 5.8 | 5.8 – 6.3 |
| --- | --- | --- | --- | --- | --- | --- | --- | --- | --- |
EPclin
groups
### Chart
| Category | 1.32867-1.82867 | 1.82867-2.32867 | 2.32867-2.82867 | 2.82867-3.32867 | 3.32867-3.82867 | 3.82867-4.32867 | 4.32867-4.82867 | 4.82867-5.32867 | 5.32867-5.82867 | 5.82867-6.32867 |
|---|---|---|---|---|---|---|---|---|---|---|
| <10 | 3.0 | 20.0 | 87.0 | 148.0 | 149.0 | 97.0 | 40.0 | 10.0 | 2.0 | 1.0 |
| 10.1-20 | 3.0 | 18.0 | 77.0 | 152.0 | 187.0 | 132.0 | 55.0 | 15.0 | 5.0 | 2.0 |
| 20.1-30 | 0.0 | 4.0 | 23.0 | 63.0 | 98.0 | 85.0 | 47.0 | 25.0 | 2.0 | 1.0 |
| 30.1-40 | 1.0 | 1.0 | 4.0 | 14.0 | 16.0 | 13.0 | 10.0 | 4.0 | 7.0 | 0.0 |
| 40.1-50 | 0.0 | 0.0 | 2.0 | 4.0 | 4.0 | 5.0 | 2.0 | 2.0 | 1.0 | 0.0 |
| 50.1-80 | 0.0 | 0.0 | 1.0 | 1.0 | 1.0 | 4.0 | 2.0 | 2.0 | 0.0 | 0.0 |
proportion of patients
EPclin high
p<0.001
EPclin low
EPclin
Ki67
Fig.3: Grouped EPclin distribution in Ki67 groups. Proportion of low risk patients are marked in blue, propotion of high risk scores are marked in red. EPclin groups estimated from EPclin cut-off 3.32867.
