# Supplementary material for: Comparison of risk assessment in 1652 early ER positive, HER2 negative breast cancer in a real-world data set: classical pathological parameters vs. 12-gene molecular assay (EndoPredict)
Source: Breast Cancer Res Treat. 2021 Nov 16;191(2):327–33. doi: 10.1007/s10549-021-06415-0 (PMC8763835; doi:10.1007/s10549-021-06415-0)
Supplement: Supplementary file 2 — Supplementary file2 (DOCX 46 kb) [file 10549_2021_6415_MOESM2_ESM.docx]

# Supplementary TABLES

**Suppl. Table 1 Comparison of latest guidelines for role of Ki67 and molecular testing for determination of adjuvant systemic therapies in ER pos, HER2 neg early BC**

| **recommendations/ guidelines for role of Ki67 and molecular testing** | |
| --- | --- |
| **NICE (2019)** | - **Ki67**: no recommendations - **molecular testing**: EndoPredict only in N0 (including micrometastatic disease) BC* - to identify intermediate risk group use validated tools, such as [PREDICT](https://breast.predict.nhs.uk/tool) or Nottingham Prognostic Index   (8) |
| **ASCO (2020)** | - **Ki67**: labeling index by immunohistochemistry should not be used to guide choice of adjuvant chemotherapy (chapter 1.26) - **molecular testing**:   - N0: clinician may use Oncotype Dx 21-gene RS/  EndoPredict 12-gene risk score (chapter 1.1,1.7)   - N+ (any): clinician should not use Oncotype Dx 21-gene RS/ EndoPredict 12-gene risk score (chapter 1.2, 1.5)   - N+ (1-3), MINDACT categorization** risk high BC: use MammaPrint   (9, 10) |
| **AJCC (2018)** | - **Ki67**: no accepted cutoff definition (AJCC Level of Evidence: III) - **molecular testing**: could be used as additional factors for clinical care, no stratification for risk groups   (11) |
| **St. Gallen (2019/2021)** | - **Ki67**: intermediate risk group is defined as 5.1 - 30% Ki67 (only for T1-2, N0-1BC) - **molecular testing**: is recommended in T1/T2 N0tumors, T3 N0 tumors, and TxN1 (one to three positive LN) BC   (23, 24) |
| **S3 (2020)** | - **Ki67:** intermediate risk group is defined between 10.1% - 25% Ki67 - **molecular testing**: could be used in N0 BC after Ki67 assessment, without clearly decision for adjuvant systemic chemotherapy (evidence level 2b)   (S3-guideline, version 4.3-2020***) |
| **ASV (2015)** | - **Ki67 & molecular testing**: Ki67 should be assessed in pathological diagnostic, and molecular test can be used in   - N0, G2 tumors or Ki67 between 10.1% and 30% but not G3 or Ki67>30%   - N+ (1-3) tumors: G1 or G2 or Ki67 between 10.1 and 30% but not G3 or Ki67>30%   (§ 116b SGB V 22.01.2015. BAnz AT 09.08.2016 B1 Bundesanzeiger****) |
| **ESMO (2019)** | - **Ki67**: intermediate risk group is defined as 10 - 30% Ki67 - **molecular testing**: should be included in pathological assessment   (1) |
| **Oncopedia (2021)** | - **Ki67**: intermediate risk group is defined as 10 - 30% Ki67 - **molecular testing**: could be used in N0-1 BC after Ki67 assessment, without clearly decision for adjuvant systemic chemotherapy   (Oncopedia*****) |
| **Ki67 working group (2020)** | - **Ki67**: intermediate risk group is defined as 5.1 - 30% Ki67 (only for T1-2, N0-1BC) - **molecular testing**: no recommendation   (12) |

*https://www.nice.org.uk/guidance/dg34

**MINDACT categorization uses validated risk tools as Adjuvant! Online and [PREDICT](https://breast.predict.nhs.uk/tool) (25);

***https://www.leitlinienprogramm-onkologie.de/fileadmin/user_upload/Downloads/Leitlinien/Mammakarzinom_4_0/Version_4.3/LL_Mammakarzinom_Langversion_4.3.pdf;

****§ 116b SGB V 22.01.2015. BAnz AT 09.08.2016 B1 Bundesanzeiger <https://www.bundesanzeiger.de/pub/de/amtliche-veroeffentlichung?4>

*****https://www.onkopedia.com/de/onkopedia/guidelines/mammakarzinom-der-frau/@@guideline/html/index.html#ID0EYYAE

**Suppl. Table 2 Comparison of Ki67 groups with EP test results from other groups**

| **publication** | **EPclin risk groups** | **Distribution of Ki-67 groups*** |
| --- | --- | --- |
| **Jank et al. (2021) N=1652** | Low risk | 4.5% with Ki67 >30% |
|  | High risk | 29.1% with Ki67 ≤10% |
| **Pellicia et al.(2021) N=100** | Low risk | 4.0% with Ki67 ≥30% |
|  | High risk | 16.7% with Ki67 <30% |
| **Noske et al. (2020) N=307** | Low risk | 10.3% with Ki67 ≥25% |
|  | High risk | 18.1% with Ki67 ≤10% |
| **Almstedt et al. (2020) N=156** | Low risk | 1.5% with Ki67 >40% |
|  | High risk | 33.7% with Ki67 <20% |

*Ki-67 groups with reverse risk status, compared to EPclin, are shown

**Suppl. Table 3 12-gene MS and EPclin comparison with classical pathological parameters**

|  | **Category** | **EPclin N (%)** | | | | **Overall N(%)** | **p-value** | |
| --- | --- | --- | --- | --- | --- | --- | --- | --- |
|  |  | **low (<3.3)** | | **high (≥3.3)** | |  |  | |
|  | overall | 626 (37.9) | | 1026 (62.1) | | 1652 (100.0) |  | |
| **Ki67 two groups** | low ≤20 | 508 (42.2) | | 695 (57.8) | | 1203 (100.0) | <0.001 | |
|  | high >20 | 118 (26.3) | | 331 (73.7) | | 449 (100.0) |  | |
| **Ki67 three groups** | low ≤10 | 258 (46.3) | | 299 (53.7) | | 557 (100.0) | <0.001 | |
|  | intermediate 10.1 - 30 | 340 (34.2) | | 654 (65.8) | | 994 (100.00) |  | |
|  | high >30 | 28 (27.7) | | 73 (72.3) | | 101 (100.0) |  | |
| **Grading** | G1 | 59 (42.1) | | 81 (57.8) | | 140 (100.0) | 0.001 | |
|  | G2 | 521 (39.2) | | 807 (60.8) | | 1328 (100.0) |  | |
|  | G3 | 46 (25.0) | | 138 (75.0) | | 184 (100.0) |  | |
|  |  | **12-gene MS N (%)** | | | | **Overall N(%)** | **p-value** | |
|  |  | **low (<5.0)** | | **high (≥5.0)** | |  |  | |
|  | overall | 410 (24.8) | | 1242 (75.2) | | 1652 (100.0) |  | |
| **Ki67 two groups** | low ≤20 | 369 (30.7) | | 834 (69.3) | | 1203 (100.0) | <0.001 | |
|  | high >20 | 41 (9.1) | | 408 (90.9) | | 449 (100.0) |  | |
| **Ki67 three groups** | low ≤10 | 225 (40.4) | | 332 (59.6) | | 557 (100.0) | <0.001 | |
|  | intermediate 10.1 - 30 | 176 (17.7) | | 818 (82.3) | | 994 (100.0) |  | |
|  | high >30 | 9 (8.9) | | 92 (91.1) | | 101 (100.0) |  | |
| **Grading** | G1 | 59 (42.1) | | 81 (57.9) | | 140 (100.0) | <0.001 | |
|  | G2 | 336 (25.3) | | 992 (74.7) | | 1328 (100.0) |  | |
|  | G3 | 15 (8.2) | | 169 (91.8) | | 184 (100.0) |  | |
|  |  | **Grading** | | | | **Overall N(%)** | **p-value** | |
|  |  | **G1** | **G2** | | **G3** |  |  | |
| **Ki67 two groups** | low ≤20 | 120 (10.0) | 996 (82.8) | | 87 (7.2) | 1203 (100.0) | <0.001 | |
|  | high >20 | 20 (4.5) | 332 (73.9) | | 97 (21.6) | 449 (100.0) |  | |
| **Ki67 three groups** | low ≤10 | 77 (13.8) | 457 (82) | | 23 (4.1) | 557 (100.0) | <0.001 | |
|  | intermediate 10.1 - 30 | 59 (5.9) | 803 (80.8) | | 132 (13.3) | 994 (100.0) |  | |
|  | high >30 | 4 (4.0) | 68 (67.3) | | 29 (28.7) | 101 (100.0) |  | |
| *****percent values determined within categorical groups | | | | | | | |  |

**Suppl. Table 4 Comparison of tumor grading vs. 12-gene MS and EPclin as continuous parameter**

|  | **Category** | **N (%)** | **mean** | **95%CI** | **t-test** | **p-value** |
| --- | --- | --- | --- | --- | --- | --- |
| **12-gene MS** | G1 | 140 (8.5) | 5.64 | 5.31-5.96 | G1 vs G2 | <0.001 |
|  | G2 | 1328 (80.4) | 6.56 | 6.44-6.81 | G2 vs G3 | <0.001 |
|  | G3 | 184 (11.1) | 8.19 | 7.85-8.54 | G1 vs G3 | <0.001 |
| **EPclin** | G1 | 140 (8.5) | 3.50 | 3.40-3.61 | G1 vs G2 | 0.324 |
|  | G2 | 1328 (80.4) | 3.56 | 3.53-3.61 | G2 vs G3 | <0.001 |
|  | G3 | 184 (11.1) | 3.87 | 3.76-4.00 | G1 vs G3 | <0.001 |
